# Field Validity and Feasibility of Four Techniques for the Detection of *Trichuris* in Simians: A Model for Monitoring Drug Efficacy in Public Health?

**DOI:** 10.1371/journal.pntd.0000366

**Published:** 2009-01-27

**Authors:** Bruno Levecke, Nathalie De Wilde, Els Vandenhoute, Jozef Vercruysse

**Affiliations:** Department of Virology, Parasitology & Immunology, Faculty of Veterinary Medicine, Ghent University, Merelbeke, Belgium; Swiss Tropical Institute, Switzerland

## Abstract

**Background:**

Soil-transmitted helminths, such as *Trichuris trichiura*, are of major concern in public health. Current efforts to control these helminth infections involve periodic mass treatment in endemic areas. Since these large-scale interventions are likely to intensify, monitoring the drug efficacy will become indispensible. However, studies comparing detection techniques based on sensitivity, fecal egg counts (FEC), feasibility for mass diagnosis and drug efficacy estimates are scarce.

**Methodology/Principal Findings:**

In the present study, the ether-based concentration, the Parasep Solvent Free (SF), the McMaster and the FLOTAC techniques were compared based on both validity and feasibility for the detection of *Trichuris* eggs in 100 fecal samples of nonhuman primates. In addition, the drug efficacy estimates of quantitative techniques was examined using a statistical simulation. *Trichuris* eggs were found in 47% of the samples. FLOTAC was the most sensitive technique (100%), followed by the Parasep SF (83.0% [95% confidence interval (CI): 82.4–83.6%]) and the ether-based concentration technique (76.6% [95% CI: 75.8–77.3%]). McMaster was the least sensitive (61.7% [95% CI: 60.7–62.6%]) and failed to detect low FEC. The quantitative comparison revealed a positive correlation between the four techniques (Rs = 0.85–0.93; *p*<0.0001). However, the ether-based concentration technique and the Parasep SF detected significantly fewer eggs than both the McMaster and the FLOTAC (*p*<0.0083). Overall, the McMaster was the most feasible technique (3.9 min/sample for preparing, reading and cleaning of the apparatus), followed by the ether-based concentration technique (7.7 min/sample) and the FLOTAC (9.8 min/sample). Parasep SF was the least feasible (17.7 min/sample). The simulation revealed that the sensitivity is less important for monitoring drug efficacy and that both FLOTAC and McMaster were reliable estimators.

**Conclusions/Significance:**

The results of this study demonstrated that McMaster is a promising technique when making use of FEC to monitor drug efficacy in *Trichuris*.

## Author Summary

Worldwide, millions of people are infected with soil-transmitted helminths, particularly in developing countries. Efforts to control these infections involve periodic mass drug treatment in endemic areas. Since these large-scale interventions are likely to intensify, monitoring of drug efficacy has become a key issue in order to detect the emergence of resistance. At present, the drop in infection intensity is under examination for monitoring the drug efficacy. However, studies comparing detection techniques based on infection intensities are scarce. Moreover, little attention has been addressed to their feasibility and their ability to estimate drug efficacies. We have compared different techniques for the detection of whipworm (*Trichuris*) in simian stool samples based on prevalence, infection intensities, feasibility and ability to estimate the ‘true’ drug efficacy. We have found that techniques often fail to detect low infection intensities and that not all techniques are appropriate for estimating infection intensities. The time needed to obtain a test result varied from 3.9 to 17.7 min/sample. Finally, accurate estimates of drug efficacy were only obtained in high pre-drug administration infection intensities. To conclude, along with accurate estimates of infection intensities, feasibility is a considerable criterion for the detection techniques used in drug efficacy monitoring programs.

## Introduction

Worldwide, infections with soil-transmitted heminths (STHs) such as *Trichuris trichiura*, *Ascaris lumbricoides* and hookworms (*Ancylostoma duodenale* and *Necator americanus*) are of major importance for public health, particularly in tropical and subtropical countries where climate factors combined with poor environmental, domestic and personal hygiene ease transmission [1],[2]. Current efforts to control STH infections involve periodic mass drug treatment of people, particularly of school-aged children, in all endemic areas [3]. Since these large-scale interventions are likely to intensify as more attention is addressed to the importance of these neglected diseases [4], monitoring drug efficacy will become indispensable in order to detect the emergence of resistance [5],[6] and/or identify confounding factors affecting the drug efficacy [7]. Thus far, the efficacy of anthelmintics has mostly been monitored qualitatively based on the cure rate. However, the fecal egg count reduction test (FECRT) is presently under examination for monitoring the drug efficacy quantitatively [8], implying the need for a sensitive detection technique which will allow the accurate estimation of the infection intensity based on fecal egg counts (FEC). Various techniques have been used for the detection of STH eggs, yet studies comparing detection techniques based on FEC are scarce. Moreover, little attention has been addressed to their feasibility for mass diagnosis under field conditions (poorly equipped laboratories and short of professionally trained personnel) and their ability to estimate the efficacy of administered drugs, in particular in different settings of pre-drug administration infection intensities.

The ether-based concentration method [9] and the Kato-Katz thick-smear technique [10] are the most commonly used techniques. The latter was initially designed for the diagnosis of *Schistosoma* eggs and not for STH such as *Trichuris*, *Ascaris* and hookworms. Due to the lack of other quantitative techniques and the importance of *Schistosoma* in public health, this method became also commonly used for the detection of STH [11]. However, this has important drawbacks when the objective of a study is to examine STH simultaneously. The most important one is the diverse clearing time of the different eggs of STH, eggs of hookworms in particular which impedes further standardization of this technique in large-scaled studies at different study sites [12],[13]. Utzinger et al. (2008) [14] recently evaluated the FLOTAC technique for the diagnosis of hookworms in stools of African schoolchildren. This quantitative technique, which has been recently described both for human and veterinary medicine [15], proved to be more sensitive than the traditionally used techniques. Other candidate techniques for monitoring drug efficacy are the McMaster technique, the precursor of FLOTAC, and the Parasep Solvent Free (SF). McMaster is a quantitative flotation technique which is commonly used in veterinary parasitology and can be easily performed without a centrifuge apparatus [16]–[18]. Parasep SF is a single use, disposable enclosed concentration technique recently developed by DiaSys Europe. In contrast to the traditional ether-based concentration technique, a fat dispersion chamber is used to separate the fat content, therefore reducing the need for chemical reagents in the field.

A study was conducted to evaluate the performance of different techniques for monitoring drug efficacy in *Trichuris*. To this end, the ether-based concentration technique, the Parasep SF, the FLOTAC and the McMaster were compared for the detection of *Trichuris* eggs in stool of nonhuman primates, focusing on validity, feasibility under field conditions and ability to estimate the ‘true’ drug efficacy using a statistical simulation. Non-human primates are an appropriate model, since these animals share the same STH and have a comparable fecal composition [19],[20].

## Materials and Methods

### Study design

The study was conducted at a Dutch sanctuary for exotic animals. All nonhuman primates involved belonged to the family of Old World monkeys and were representatives of barabary macaques (*Macaca sylvanus*), vervet monkeys (*Chlorocebus pygerythrus*), rhesus monkeys (*Macaca mulatta*), crab-eating macaques (*Macaca fascicularis*), Sunda pig-tailed macaques (*Macaca nemestrina*), grivet monkeys (*Chlorocebus aethiops*) and Hamadryas baboons (*Papio hamadryas*). The animals were housed in 22 groups of one to 15 animals. A total of 100 fecal samples were randomly collected by the animal caretakers on a single occasion in January 2008.

### Detection of *Trichuris* eggs

Three grams of feces were suspended in distilled water and strained through a layer of surgical gauze to withhold large debris. After sedimentation for 1 h and centrifugation at 800 *g* for 5 min, the sediment was suspended in 3 ml of distilled water. An aliquot of 500 µl was randomly assigned to each of the four detection techniques.

#### Ether-based concentration technique

The aliquots were suspended in 5 ml acetic acid (5%). The fat in the suspension was removed by emulsifying the sample with an equal volume of ether followed by centrifugation at 200 *g* for 2 min. The resulting supernatant (ether, debris and acetic acid) was discarded and 2 drops of diluted (1∶10) iodine were added to the remaining sediment. The stained sediment was thoroughly mixed, transferred onto a glass microscope slide and covered with a cover glass. Each sample was examined at a 100× magnification. The FEC were multiplied by 2 to obtain the number of eggs per gram of faeces (EPG) [21].

#### Parasep SF

The Parasep SF was performed as described by the manufacturer (DiaSys Europe, Berkshire, England). The aliquots were added to 8 ml of 10% formalin and one drop of surfactant (Triton X-100) in the Parasep SF device. The suspension was well shaken and centrifuged at 200 *g* for 3 min. The supernatant was discarded and the sediment was processed by analogy with the ether-based concentration technique.

#### McMaster technique

The aliquots were filled up to a volume of 7.5 ml with saturated salt and sucrose solution (density = 1.22), stirred thoroughly, and then 0.15 ml amounts were added to each of the 2 chambers of a McMaster slide. Both chambers were examined using a 100× magnification and the FEC was obtained by multiplying the total number of eggs by 50 [21].

#### FLOTAC technique

A volume of 5 ml of a sucrose solution (density = 1.27) was added to the aliquots. The suspension was pipetted in one of the 2 flotation chambers of the FLOTAC apparatus. After centrifugation of the apparatus at 200 *g* for 5 min, the apical portion of the floating suspension was translated (top of the flotation suspensions was cut off transversally), followed by examining both grids corresponding to 2 different samples at a magnification of 100×. The total number of eggs was multiplied by a factor of 2 to calculate the FEC [15].

### Assessment of the feasibility of the techniques

The feasibility of the 4 the techniques was evaluated on a total number of 90 samples randomly assigned to 3 experienced laboratory technicians. Each of the laboratory technicians processed a set of 1, 2, 4 and 8 samples. The time needed to prepare the samples and to clean the devices was measured six times for each set of samples. The preparation period started when the aliquots were distributed and ended when the sample was ready for examination. During the cleaning period all used materials were either disposed of, for single use components, or cleaned in the case of recyclable devices. Examination of the slides or chambers was timed individually for all samples. Each of the samples were examined with all techniques by the same laboratory technician.

### The estimation of the ‘true’ drug efficacy

The estimates of the ‘true’ drug efficacies (TDE) by the quantitative techniques was studied in a statistical simulation in R (version 2.4.0, The R Foundation for Statistical Computing). To this end, difference between the TDE and the estimated drug efficacy (EDE) was examined in two strata with different pre-drug administration infection intensities. In this simulation, the TDE for *Trichuris* varied from 10 to 90% [22]. Because these values are likely to vary between individuals (‘individual’ TDE) an additional variation was postulated for each value of TDE, ranging from 1 to 5% (standard deviation) [7]. Only the positive samples of the quantitative techniques in the present study (a positive result at any technique) were included as pre-drug administration samples. These samples were stratified into a stratum with low pre-drug administration intensity (0<FEC≤50 EPG) and one with a high pre-drug administration intensity (FEC>50 EPG). The cut off of 50 EPG was used, since this was the highest detection limit of all the techniques. The samples in each strata were combined into 4 different sample sets of 100, 250, 500 and 1000 samples to examine the effect of sample size on the efficacy estimates. The individual post-drug administration infection intensities for each technique were obtained by multiplying the individual pre-drug administration FEC of each technique with *(100%-individual TDE)*. The obtained individual post-drug administration FEC of each technique were corrected according the results of the validity. The estimated efficacy of each individual for each technique was calculated as the difference of individual pre- and post-drug administration FEC divided by the individual pre-drug administration FEC obtained by each technique. The difference between the TDE and the individual EDE (bias) for each detection technique, standard deviation, sample set was examined within each stratum of pre-drug administration infection intensities.

### Statistical analysis

To assess the validity of the techniques both qualitative and quantitative aspects were compared. The agreement in qualitative test results between the 4 techniques was measured using the kappa statistic (κ) (SAS 9.1.3, SAS Institute Inc.; Cary, NC, USA). Both sensitivity and negative predictive value (NPV) were estimated for each method. To this end, a positive result at any technique was considered as a ‘true’ positive test result (specificity = 1, positive predictive value = 1). The agreement in quantitative test results was estimated by the Spearman rank correlation coefficient (SAS 9.1.3, SAS Institute Inc.; Cary, NC, USA). In addition, the Wilcoxon signed rank test was assessed to test for differences in FEC between the techniques. For this end, a Bonferroni pair-wise comparison procedure was performed and the level of significance was set at 0.0083. Furthermore, samples were subdivided according infection intensity based on guidelines of the WHO [23]. A linear mixed model was built using the Tukey pair-wise comparison test to evaluate the feasibility of the 4 techniques (SAS 9.1.3, SAS Institute Inc.; Cary, NC, USA). This model examined the differences in time taken for preparing and cleaning a sample between the different sample sets and the 4 techniques. The differences in time taken for the microscopic examination of the samples prepared by the 4 techniques and the effect of FEC on time taken were studied separately.

### Ethical considerations

This study was embedded in a annual parasitological survey of the animals housed at the sanctuary for exotic animals AAP. No approval of the Ethics Committee was needed since all samples were randomly collected during cleansing of the enclosures and none of the animals involved were immobilized neither physically or chemically (European Convention for the Protection of Vertebrate Animals used for Experimental and Other Scientific Purposes, Strasbourg, 18.III.1986).

## Results

### Technique validity

#### Agreement in qualitative results

Of the 100 samples examined by the 4 different diagnostic assays, there were 30 positive based on the McMaster, 36 for the ether technique, 39 for the McMaster and 47 using the FLOTAC. There was a full agreement, either positive or negative, among all four tests in 82 samples. The observed agreement between the four tests varied from substantial (κ = 0.65, [95% confidence interval (CI): 0.73–0.94]) to almost perfect (0.89 [95% CI: 0.80–0.98]). The least agreement was found between McMaster and FLOTAC, the most between the ether technique and the Parasep SF. The test properties of the techniques are summarized in Table 1.

**Table 1:** Test properties of the FLOTAC, the Parasep SF, the ether and the McMaster technique.

	Number of positive samples	Sensitivity (%) (95% CI)	NPV (%) (95% CI)*
FLOTAC	47	100	100
Parasep SF	39	83.0 (82.4–83.6)	90.0 (84.9–95.5)
Ether	36	76.6 (75.8–77.3)	88.1 (82.4–93.8)
McMaster	30	61.7 (60.7–62.6)	85.6 (79.5–91.8)

NPV: negative predictive value;

*: based on Monte Carlo simulation.

#### Agreement in quantitative test results

The median FEC and the distribution of 3 different levels of infection intensity (low, moderate and heavy) for each of the 4 techniques are summarized in Table 2. The median value was the highest for the McMaster technique, but comparable to the median of FLOTAC.

**Table 2:** Median (25^th^–75^th^ quartile) of FEC and the distribution of 3 different levels of infection intensity (95% CI) detected by the FLOTAC, the Parasep SF, the ether and the McMaster technique.

	Median (EPG) (25^th^–75^th^ quartile)	Low (%) (95% CI) (0<FEC<1000)	Moderate (%) (95% CI) (1000≤FEC<10 000)	Heavy (%) (95% CI) (10 000≤FEC)
FLOTAC	66 (10–946)	76.6 (64.5–88.7)	23.4 (11.3–35.5)	0
Parasep SF	12 (2–90)	94.9 (88.0–100)	5.1 (0–12.0)	0
Ether	14 (2–132)	100	0	0
McMaster	100 (0–700)	70.0 (53.6–86.4)	30.0 (13.6–46.4)	0

**Figure 1:**
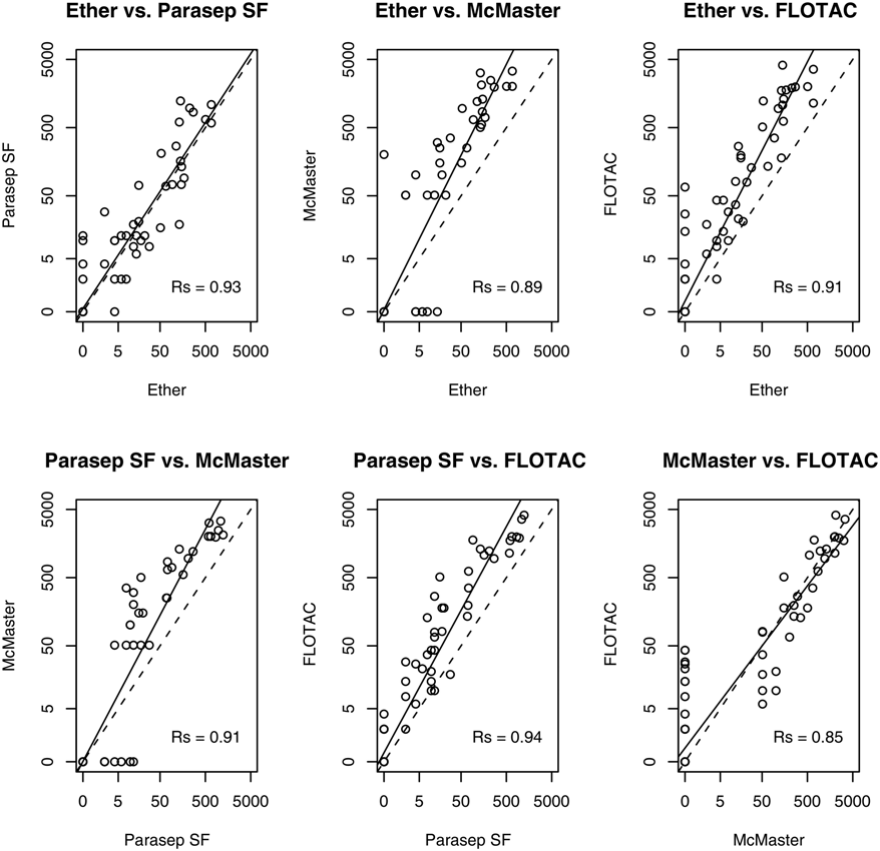
Pair-wise comparison of the 4 techniques based on FEC.

Overall, there was a significant (*p*<0.001) linear correlation (Rs) in FEC between the 4 techniques, ranging from 0.85 to 0.94 (Figure 1). Although these Rs values were comparable, the concordance plots clearly illustrate a difference in level of agreement between the techniques. Only the FEC between the ether and the Parasep SF technique and between the McMaster and the FLOTAC were scattered around the dashed equality line (slope = 1). Other pair-wise comparisons showed a slope greater than 1, indicating that the technique in the x-axis is detecting fewer eggs than the technique in the y-axis. Both the ether and the Parasep SF techniques detected significant less eggs compared to McMaster and FLOTAC (*p*<0.0083). These plots also indicate that both the ether technique and the McMaster often fail to detect FEC of less than 50 EPG, while the Parasep SF failed to detect eggs in some samples with less than 5 EPG.

### Technique feasibility

#### Preparation and cleansing of the samples

**Figure 2:**
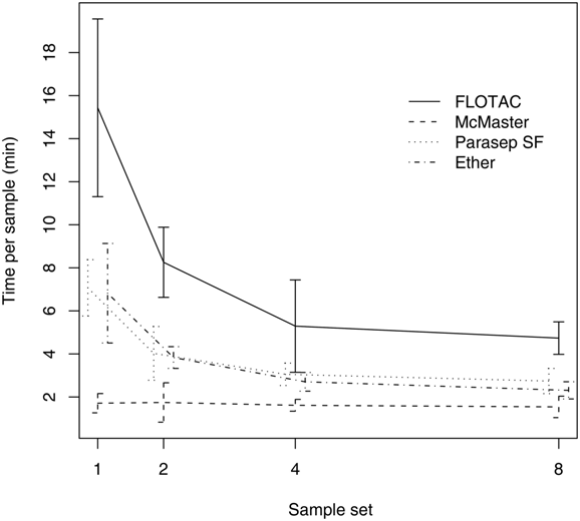
Mean time per sample (combined preparation and cleaning) and 95% CI for the 4 different techniques and the 4 sample sets.

Overall, the most time consuming method was FLOTAC (mean time 8.4 min/sample), followed by the ether (4.2 min/sample) and the Parasep SF technique 4.0 min/sample). McMaster (1.6 min/sample) was the least time consuming. Figure 2 describes the time needed to prepare and clean sets of 1, 2, 4 and 8 samples for each of the 4 techniques. Increasing the sample sizes significantly decreased the time per sample for FLOTAC, the ether and the Parasep SF technique. The most benefit was gained between sample sets of one and 2 samples, but there was also a decrease in average time between 2 and 4 samples. No significant beneficial effect was found between the sample sets of 4 and 8 samples. For the McMaster no significant difference between sample sets was found. The time period for preparation and cleaning of sample sets of 4 and 8 was equivalent for the ether, the Parasep SF and the McMaster techniques.

#### Examination of the samples

**Figure 3:**
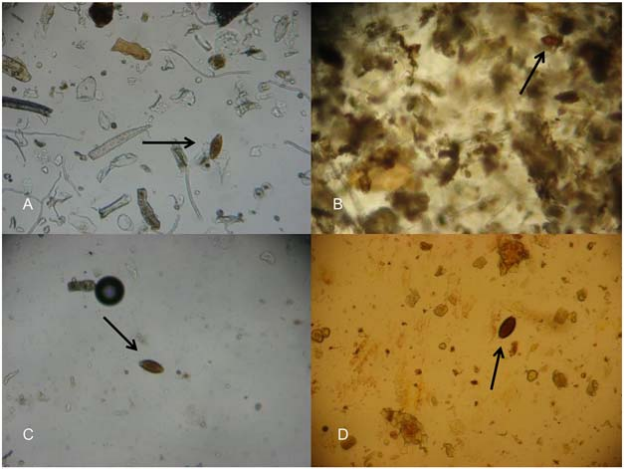
Appearance of microscope slides loaded with egg samples prepared by the ether technique (A), the Parasep SF (B), the McMaster (C) and the FLOTAC (D). Arrows indicate *Trichuris* eggs.

Samples prepared by the Parasep SF (14.0 min/sample) were the slowest to examine, followed by the ether technique (5.4 min/sample) and the FLOTAC (5.1 min/sample). The McMaster slides (2.4 min/sample) were the quickest to read. Higher FEC for a particular sample significantly increased (*p*<0.001) the time for examining the sample for all techniques, except for McMaster. Figure 3 illustrates the visibility of the samples for the 4 detection techniques. It is apparent that the eggs were much more clearly visible for the McMaster samples, followed by approximate equivalence in the FLOTAC and the ether samples. Samples prepared by the Parasep SF revealed a high level of contamination with fecal debris.

Combining the results of the preparation, examination and cleaning for the largest sample set (8 samples), revealed that McMaster was the most feasible of all the techniques. On average, a test result was achieved within 4 minutes (mean time 3.9 min/sample). The ether technique (7.7 min/sample) and the FLOTAC (9.8 min/sample) were comparable in feasibility. The Parasep SF was the least feasible, as almost 18 min (mean 17.7 min/sample) was required for complete processing of the sample.

### The estimation of the ‘true’ drug efficacy

Based on FLOTAC, a total of 47 positive samples of which 23 had a FEC of no more than 50 EPG were included in the simulation. Additional samples were randomly chosen to add to the strata (2 for the low pre-drug administration infection intensity stratum, 1 for the high pre-drug administration infection intensity stratum), resulting in two subsets of each 25 samples. Each subset was combined 4, 10, 20 and 40 times to obtain the different sample sets of 100, 250, 500 and 1000, respectively. Both the ether technique and Parasep SF were withdrawn for further analysis, since these detection techniques resulted in significant lower FEC compared to FLOTAC and McMaster. Based on the results of the validity for McMaster, the individual post-treatment FEC were corrected using the following conditions. At first, all positive post-drug administration samples which had no more than 5 EPG were negative for McMaster. Samples with a FEC of more than 5 EPG, but no more than 50, were positive with a probability of 0.40 and the FEC was set on 50 EPG if positive. The FEC was rounded off to the nearest multiple of 50, in all other cases. Although FLOTAC revealed to be 100% sensitive, positive samples with no more than 2 EPG were positive with a probability of 0.40 and FEC was set on 2 EPG if positive. In all other cases, the FEC was rounded off to the nearest multiple of 2.

**Figure 4:**
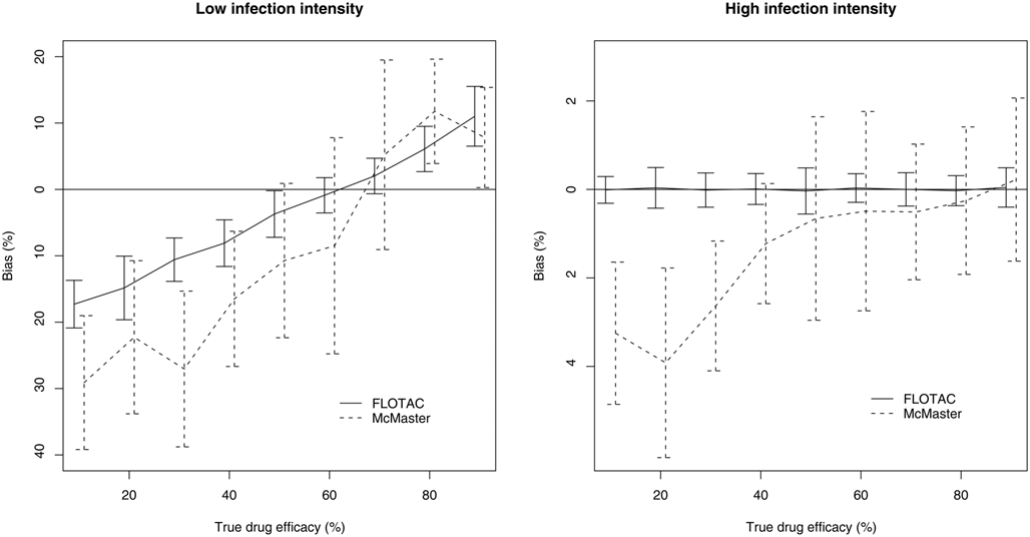
Mean bias and 95% CI of FLOTAC and McMaster for different TDE in 2 strata of different pre-drug administration infection intensities.

Figure 4 describes the bias (difference between TDE and the individual EDE) in 2 strata with different pre-drug administration infection intensities. Overall, there is a large bias in estimating the drug efficacy when low pre-drug administration infection intensities were included. The mean bias in the stratum including FEC not higher than 50 EPG ranged from −20.8 to +16.1% for FLOTAC and from −45.0 to +20.0% for McMaster. Moreover, this bias is likely to change over TDE. Low TDE (≤60.0%) were overestimated and high TDE (>60.0%) were underestimated. This is in contrast to the stratum where only FEC higher than 50 EPG were considered, in particular for FLOTAC which resulted in an accurate estimation of the TDE (median = 0.0%, range = −0.6; +0.7%). The differences for McMaster varied from −6.4 to +2.3% (median = −1.1%), and decreased with the TDE-values of at least 50% (median = −0.3%, range = −3.6; +2.1%). In average, McMaster resulted in higher efficacies than FLOTAC, since the bias was more negative than the efficacy estimated by FLOTAC in the majority of the ‘true’ drug efficacies in both strata.

**Figure 5:**
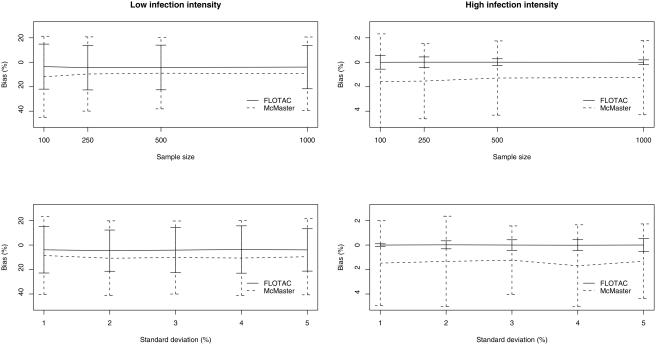
Mean bias and 95% CI of FLOTAC and McMaster for different sample sizes and standard deviations in 2 strata of different pre-drug administration infection intensities.

Both the different standard deviations and sample sets did not affect the estimates of both techniques (Figure 5), since the bias remained unchanged over the different standard deviations and sample sets.

## Discussion

In the present study, four techniques were compared for the qualitative and quantitative detection of *Trichuris* in stools of nonhuman primates, as well as their feasibility for mass diagnosis under field conditions. In addition, their ability to give accurate estimates of the ‘true’ drug efficacy was studied based on a statistical simulation. Overall, the observed prevalence of *Trichuris* in these animals was 47% (95% CI: 37–57%) and remained unchanged when the test results of the 4 techniques were combined. Although the test properties might be overestimated due to the absence of a diagnostic ‘gold’ standard, it is clear that FLOTAC is the most sensitive technique, followed by the Parasep SF and the ether-based concentration technique. McMaster is the least sensitive, because it often fails to detect low FECs due to its relative high detection limit (50 EPG). Multiple comparisons of the 4 techniques revealed a linear correlation in FEC. Nevertheless, both the Parasep SF and ether techniques are likely to be less appropriate for an accurate estimation of FEC, since the McMaster and the FLOTAC detected significantly more eggs.

The time taken for preparing samples and cleaning between samples was the lowest for the McMaster. FLOTAC was the most time- and labour-consuming technique over the different sample sets and therefore seems to be less feasible in large-scale studies. Samples obtained by the Parasep SF protocol were the hardest to examine, because of the large amount of fecal debris recovered with the eggs. This resulted in decreased clarity and a much greater length of time required to examine the samples. McMaster slides were examined at the highest speed. The most important reasons explaining the difference in time required for reading between McMaster and the other techniques are the surface of the slides (McMaster: 1 cm^2^ versus other techniques: 3.24 cm^2^) and the detection limit (McMaster: 50 versus other techniques: 2), as FEC had no significant effect on the time needed to examine the samples using McMaster. Moreover, McMaster slides were likely to be less contaminated by fecal debris, since only a small proportion of already diluted samples was examined. Overall, McMaster was the most feasible and does not need any centrifuge apparatus, in contrast with the other techniques, which clearly emphasizes its usefulness in poorly equipped and often short-staffed laboratories.

Estimating drug efficacies should be done on samples with high FEC (>50 EPG), as including samples with lower FEC may result in a significant bias. As a consequence, sensitivity as a criterion for detection techniques for monitoring drug efficacy is less important. FLOTAC resulted in the most accurate estimates of TDE in the stratum of high FEC. However, the bias when using McMaster is minimal (median = −1.1%) and is comparable to FLOTAC if the TDE (median = −0.3%) is at least 50%.

Although the results presented in this study were obtained from stool of nonhuman primates, these will also be applicable in human parasitology. These animals not only share the same STH species with humans, they also have a similar fecal composition [19],[20]. Moreover, the prevalence of *Trichuris* is comparable to those of previous epidemiological studies in pre-school children, where in average 39% of the subjects were infected [3]. Furthermore, the distribution of the FEC found in these animals was similar. Based on WHO guidelines, 23.4% (FLOTAC) to 30.0% (McMaster) of the *Trichuris* infections fell into the moderate FEC range, where this was roughly 25% in Zanzibari infants [13].

Not including the Kato-Katz method in the present study is a major shortcoming. However, the present study suggests that McMaster is likely to be more feasible. The microscopic view is clear and all parasites can be examined simultaneously, which is in contrast to the Kato-Katz technique due to a different clearing time of the different STH. Based on previous study where both FLOTAC and Kato-Katz were compared for the detection of hookworm [14], we expect that both the sensitivity and the FEC of the Kato-Katz will be comparable to McMaster.

McMaster is commonly used for both diagnosis and drug efficacy monitoring programs of gastrointestinal parasites in livestock, including *Ascaris* and hookworm [16]–[18],[24], but its usefulness in detecting these STH in public health still needs to be confirmed by further studies in endemic areas where also *A. lumbricoides* and hookworms are present.

In conclusion, this study indicates that McMaster holds promise as the method of choice for monitoring drug efficacy, since sensitivity appeared to be a less important criterion. It is a quantitative technique which can be easily performed under field conditions and gives reliable estimates of TDE which are at least 50%.
